# Describing the Stem Cell Potency: The Various Methods of Functional Assessment and *In silico* Diagnostics

**DOI:** 10.3389/fcell.2016.00134

**Published:** 2016-11-22

**Authors:** Vimal K. Singh, Abhishek Saini, Manisha Kalsan, Neeraj Kumar, Ramesh Chandra

**Affiliations:** ^1^Stem Cell Research Laboratory, Department of Biotechnology, Delhi Technological UniversityDelhi, India; ^2^Department of Chemistry, University of DelhiDelhi, India

**Keywords:** pluripotency, iPSCs, teratoma, chimera, EpiSCs, EpiBlast, naïve, primed

## Abstract

Stem cells are defined by their capabilities to self-renew and give rise to various types of differentiated cells depending on their potency. They are classified as pluripotent, multipotent, and unipotent as demonstrated through their potential to generate the variety of cell lineages. While pluripotent stem cells may give rise to all types of cells in an organism, Multipotent and Unipotent stem cells remain restricted to the particular tissue or lineages. The potency of these stem cells can be defined by using a number of functional assays along with the evaluation of various molecular markers. These molecular markers include diagnosis of transcriptional, epigenetic, and metabolic states of stem cells. Many reports are defining the particular set of different functional assays, and molecular marker used to demonstrate the developmental states and functional capacities of stem cells. The careful evaluation of all these methods could help in generating standard identifying procedures/markers for them.

## Introduction

Stem cells are unique in their capabilities to either self-renew or differentiate into various cell lineages. The most primitive stem cells have the potential to generate all the cell types in any organism and termed as pluripotent stem cells (PSCs) (Hanna J. H. et al., 2010). Whereas, multipotent and unipotent stem cells are defined to bear limited self-renewing capacity and these cells, differentiate into a particular tissue type or cell lineage. It is important to notice that cells derived from fertilized egg (zygote/blastomeres) have the potential to generate all the embryonic and extra-embryonic cells; potential referred to as totipotency and thus can give rise to the whole organism, however their developmental potential remains undefined *in vitro* (Kelly, 1977). Both human and mouse derived Embryonic Stem Cells (ESCs) are demonstrated to generate all types of cells, but lack potential to contribute to the extra-embryonic cells such as placenta. These cells are termed as PSCs and have various functional properties depending on their culture conditions. Another important class of stem cells is lineage specific multipotent stem cells [e.g., Hematopoietic Stem Cells (HSCs)] which have limited differentiation potential and develop only in their tissue/cell types. The multipotent stem cells do not differentiate into cell types of different tissue origin under normal physiological circumstances. The developmental potential of unipotent stem cells is further restricted and they remain able to give rise to only a single cell type (for example, blast forming unit-erythroid (BFU-E) may be differentiated into erythrocytes).

Thus, the traditional developmental dogma follows the differentiation of totipotent stem cells to PSCs, PSCs to multipotent stem cells, multipotent stem cells to unipotent stem cells and finally mature cells. Both the self-renewal capacity and differential potential are reduced during their journey from totipotent to mature cell state. However, the discovery of nuclear reprogramming methods such as somatic cell nuclear transfer method and use of transcriptional factors to induce pluripotency in any cell type are demonstrated as powerful tools to reverse this hierarchy (Gurdon, 1962; Kato and Tsunoda, 1993; Campbell et al., 1995, 1996; Wilmut et al., 1997; Kato et al., 1998; Wakayama et al., 1998; Wakayama and Yanagimachi, 1999; Takahashi and Yamanaka, 2006; Takahashi et al., 2007). These findings show that the particular state of a somatic cell can be reprogrammed to achieve a totipotent or pluripotent state. iPSC generated from patients have great potential in disease modeling and regenerative medicine (reviewed by Singh et al., 2015). It is clear that defining various fundamental levels of pluripotency states (e.g., naïve vs. prime etc.) remain central in developing various strategies for their clinical/research uses and therefore it is important to rigorously evaluate the different methods/molecular markers etc. reported so far for the various PSCs types.

A comprehensive review of all the functional assays defining the pluripotent states of stem cells would be of great importance to assess the functional applications and reprogramming efficiency of different methods and cell sources that are being explored both in clinical and research settings. Recently, many researchers have developed few alternative approaches such as *in silico* analysis to detect pluripotency or differentiation potential of any existing or new cell for clinical and research purposes (Sato et al., 2003; Sperger et al., 2003; Bhattacharya et al., 2004; Suárez-Farinas et al., 2005; Müller et al., 2008). It would be of great importance to have more concrete definitions and defining markers to demonstrate the significance of these *in silico* approaches and decide the clinical utility of the particular cell population that is to be used. Present article focuses on the various molecular markers and diagnostic strategies being used to define the exact state of any given cellular population that is assumed to be pluripotent or multipotent and may be used further in any relevant clinical/research regime. As discussed in the later sections, there are many molecular markers (including TFs e.g., OCT4, SOX4, NANOG etc.; micro RNAs, Transcriptional regulators and epigenetic chromosomal modifiers, etc.; discussed in detail in later sections) that are promptly used for a quick evaluation of cellular potency by most researchers/clinicians. Although, the complexity associated with the definition of the actual state of pluripotency (e.g., ground state, naïve, and prime states of pluripotency etc.) and the incapability of individual “pluripotency-defining molecular markers” which often remain doubtful, has raised the demand for identification of more conspicuous definitions and diagnostic tools. Since then, functional assays have remained the gold test for defining pluripotency of all types of stem cells in vertebrates. There are a variety of functional assays which differ both in the time and effort taken to perform them and the accuracy of the results obtained from these assays. The different studies done in mice, human, and other mammals have been useful to define the functional assay in different categories.

### Pluripotent stem cells: the definition

The standard definition of PSCs relies on the fact that they can produce all three germ cell layer cells and thus can give rise to almost all types of cells in the body (Table 1; Hanna J. H. et al., 2010). However, PSCs seem to lack capabilities of producing extra embryonic cells and thus may not contribute to the development of placental cells. These PSCs is therefore different from ***Totipotent stem cells*** *(capable of giving rise to extra-embryonic tissue also)* since they may not be developed into a complete organism. Another important feature for defining pluripotency has been their capabilities to self-renew themselves. However, recent studies revealed that PSCs can be maintained in self-renewing states *in vitro* indefinitely through a number of growth factors/exogenous signals indicating that the self renewing capacity may not be the defining hallmark of pluripotency (Nichols and Smith, 2012). Studies done by using various types of pluripotent cells indicated that pluripotency is more accurately defined as a transient and dynamic state *in vitro* and different types of PSCs can be derived from different stages of developing embryos in vertebrates. The pluripotency of these cells is reflected through the expression of TFs, assessment of genomic integrity, and their capability to differentiate robustly into all 3 cell lineages.

**Table 1:** **Differentiation of PSCs in all the three cell lineage**.

**Pluripotent cell used**	**Germ layer**	**Tissue**	**Organism**	**References**
Mouse embryonic stem (ES) cells	Endoderm	Hepatocytes	Mouse	Hamazaki et al., 2001
Murine embryonic stem (ES) cells	Endoderm	Hepatocytes	Murine	Jones et al., 2002
Embryoid bodies (EBs)	Endoderm	Hepatocytes	Mice	Yamada et al., 2002a
Embryonic stem cells	Endoderm	Early Pancreas	Human	Colman, 2004
Murine embryonic stem cells	Endoderm	Early Pancreas	Murine	Ku et al., 2004
Mouse embryonic stem (ES) cells	Endoderm	Thyrocytes	Mouse	Lin et al., 2003
Murine embryonic stem cells	Endoderm	Type II Pneumocytes	Murine	Ali et al., 2002
Mouse embryonic stem (ES) cells	Endoderm	Intestinal cells	Mouse	Yamada et al., 2002b
Human embryonic stem cells	Mesoderm	Hematopoietic	Human	Kaufman et al., 2001
Mouse yolk Sac	Mesoderm	Vascular	Mouse	Haar and Ackerman, 1971
Human embryonic stem cells	Mesoderm	Cardiac	Human	Nir et al., 2003
Mouse embryonic stem (ES) cells	Mesoderm	Cardiac	Mouse	Hescheler et al., 1997
Mouse embryonic stem (ES) cells	Mesoderm	Cardiac	Mouse	Min et al., 2002, 2003
Mouse embryonic stem (ES) cells	Mesoderm	Skeletal Muscle	Mouse	Rohwedel et al., 1994
Murine embryonic stem cells	Mesoderm	Osteogenic	Murine	Buttery et al., 2001
Mouse blastocyst	Mesoderm	Osteogenic	Mouse	Zur Nieden et al., 2003
Mouse embryonic stem (ES) cells	Mesoderm	Chrondrogenic	Mouse	Kramer et al., 2000
Murine embryonic stem cells	Mesoderm	Adipogenic	Murine	Dani et al., 1997
Mouse embryonic stem (ES) cells	Ectoderm	Neuroepithelium	Mouse	Li et al., 1998
Human embryonic stem cells	Ectoderm	Neural Cells	Human	Reubinoff et al., 2001; Zhang et al., 2001

**Figure 1:**
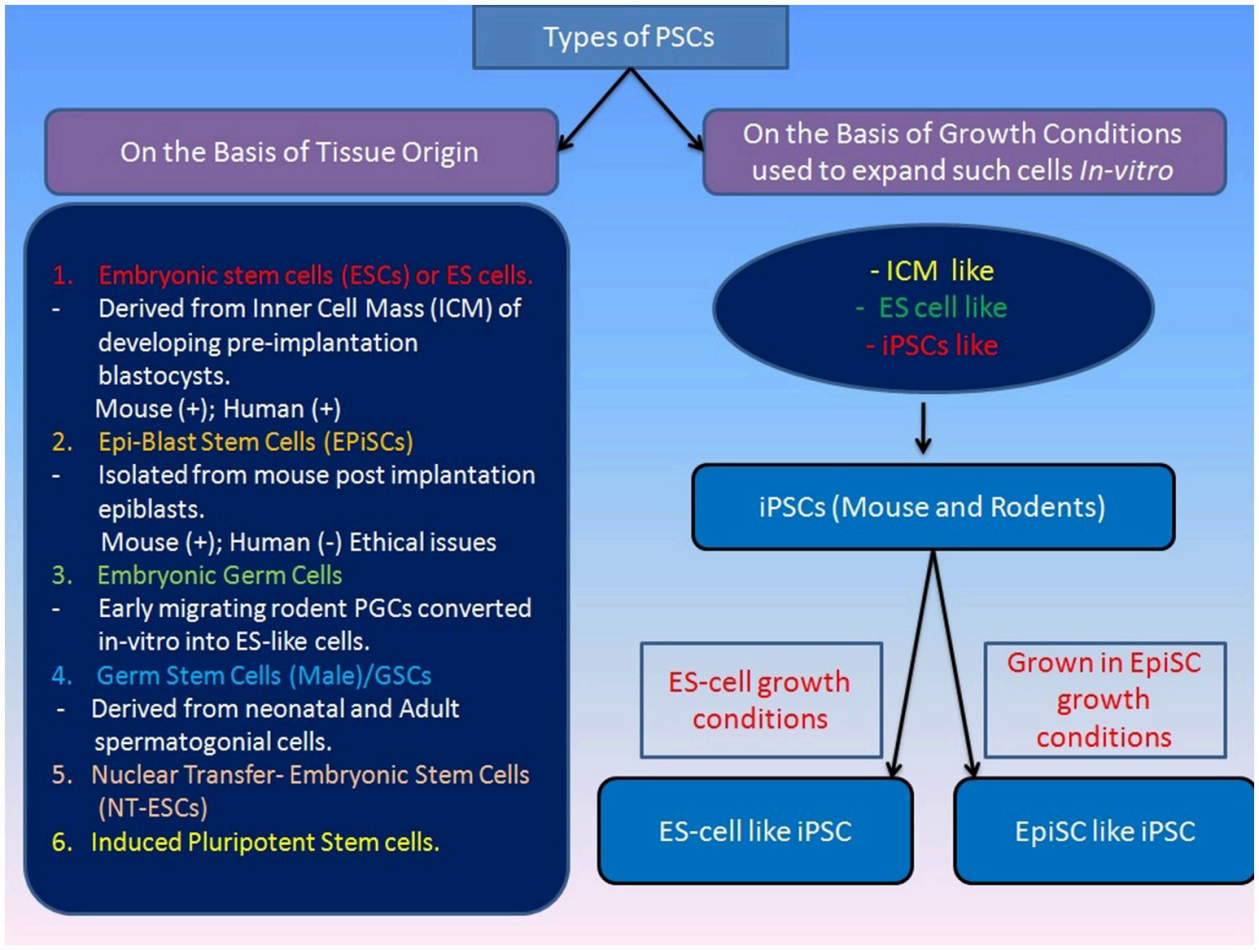
**Classification of various types of pluripotenct stem cells on the basis of their origin**. Various types of pluripotent stem cells can be classified on the basis of their origin or source from where they are taken and this would directly define their self-renewing and differentiation potential. Pluripotency of stem cells is also regulated through their culture microenvironment and they are often named on the basis of their similarity to the various types of previously established PSCs e.g., ICM-like or ES-like PSCs. [Mouse (+), Human (+) indicates derivation of ESCs inthese mammals; Human(−) indicates non-derivation of post-implantation EPIsc in humans].

There are multiple types of PSCs (including rodents, humans and primates) that are typically classified on the basis of their origin from a specific tissue (Figure 1) such as various germline tumors, blastomeres, and inner cell mass of developing embryos (Box-1). Somatic cell reprogramming techniques gave alternative tools for generating PSCs both in mouse and humans. Somatic cells have been reported to become ESCs-like by *Nuclear Transfer* resulting into NT-ESCs (Wakayama et al., 2001; Tachibana et al., 2013; Yamada et al., 2014). Similarly, PSCs can be derived by inducing reprogramming through ectopic expression of a number of different TFs (Takahashi and Yamanaka, 2006). The ectopic expression of basal reprogramming factors (OCT4, KLF4, SOX2, and c-MYC) in the mouse fibroblasts led to their reprogramming into pluripotent cells, which were called as induced pluripotent stem cells (iPSCs; Takahashi and Yamanaka, 2006). The generation of these iPSCs came out as breakthrough as it gave a way of generating PSCs from somatic cells without having any requirement of ESCs which have associated ethical concerns. Since then, researchers all over the world have used this technique to reprogramme different somatic cell types into iPSCs (Yu et al., 2007). Many groups have also reported different approaches to reprogramming, which include the exclusion or replacement of one or more of the basal 4 reprogramming factors, along with the inclusion of different small molecules that have been reported to increase the reprogramming efficiency (Feng et al., 2009). IPSCs pose to have many applications in the stem cell therapies, drug toxicity assays, disease modeling, gene therapy, and hence they hold great importance (Singh et al., 2015).

Box 1 **Multiple types of PSCs in mouse and humans**

**Mouse pluripotent stem cells**

Initially, Stevens and his colleagues demonstrated the formation of teratocarcinoma cells by using strain 129 mice model and thus *in vitro* methods for the propagation of ESCs derived from similar mice model studies was established (Stevens, 1958). Subsequently, methods for the propagation of immortalized non-transformed embryonic stem cell lines were demonstrated by growing cells derived from murine blastocysts preconditioned media with preformed teratocarcinoma stem cell line (Evans and Kaufman, 1981; Martin, 1981). This method of obtaining ESCs was followed by some researchers for the isolation of pluripotent cells lines from various types of sources (Thomson et al., 1995). Initial studies suggested that mouse ESCs could only be obtained from the embryo before implantation in the uterus. However, later reports established that rodent ESCs can be derived from the post-implantation embryo (epiblast tissue) that generates the embryo proper (Brons et al., 2007; Tesar et al., 2007). These cells are referred as EpiSCs (post-implantation epiblast-derived stem cells), and can be derived by culturing cells in chemically defined media along with other supplements (Brons et al., 2007; Tesar et al., 2007). For ethical reason similar EpiSCs are not attempted in humans so far.

Early migrating mouse PGCs are shown to have pluripotent ESC-like properties when grown *in vitro* under specific conditions and termed as embryonic germ cells (Matsui et al., 1992; Leitch et al., 2010). The mouse PGCs were grown up to 20 passages in the presence of “Steel Factor” (membrane-associated SF) and “Leukemia Inhibitory Factor” (LIF) and bFGF supporting their continued proliferation. The cells exhibit pluripotency through the generation of embryoid bodies and multiple differentiated cell phenotypes in monolayer culture and tumors in nude mice, and can also contribute to chimeras when injected into host blastocysts (Matsui et al., 1992; Leitch et al., 2010).

The successful establishment of ES-like cells has been reported from neonatal mouse testis exhibiting phenotypic similarities to ESCs/EGCs except in their genomic imprinting pattern (Kanatsu-Shinohara et al., 2004). In similar studies, adult unipotent germline stem cells (GSCs) was reported to be capable of differentiating into multiple cell types both by *in vivo* and *in vitro* assays, including germ cell contribution and transmission (Ko et al., 2009). There have been efforts to define marker profiling of ESCs and it is shown that ESCs are distinct from early inner cell mass (ICM) and closely resemble preimplantation epiblast (Boroviak et al., 2014).

Many studies have revealed manifestation of distinct features of various types of PSCs attributed to their origin and maintenance conditions (Figure 1). Depending on their origin, they are coined with different terms such as ESCs (established from pre-implanted embryos) and Epiblast stem cells or EpiSCs (generated from later embryonic epiblast stages; Figure 1; Brons et al., 2007; Tesar et al., 2007). They are also often termed as “naïve” (for ESCs) and “primed” (for EpiSCs) pluripotent stem cells to highlight their early and late phases of development (Nichols and Smith, 2009). The mouse “naïve” and “primed” ESCs differ in their capacity to form high-grade chimeras with naïve ESCs being able to form chimeras but primed ESC remain incapable. However, the difference in the chimera forming capabilities of naïve and primed PSCs could be attributed to the use of suboptimal regimes for chimera generation experiments and development of more defined protocols by addition of some regulatory factors (e.g., FGF-4) may improve the chimera forming efficiency of even primed PSCs in mouse at least (Joo et al., 2014). The conventional human PSCs (hESCs) look similar to EpiSC by their molecular markers thus called as “primed” ESCs. However, “naïve” pluripotency may not be evaluated in hESCs due to ethical restriction on the formation of human chimeras. Nevertheless, existence of a naïve state of pluripotency in hESCs has been reported recently, and it is believed that the pluripotency of hESCs also can be enhanced by genetic modification or optimized culture systems (Buecker et al., 2010; Hanna J. et al., 2010; Lengner et al., 2010; Xu et al., 2010; Gafni et al., 2013). The nonhuman primates ESCs have been assessed for naïve pluripotency showing their inability to form a chimera with pre-implantation embryos (Tachibana et al., 2012).

## Evaluation of pluripotency through molecular marker profiling and functional assays

### Development of various molecular diagnostics for PSCs

**Figure 2:**
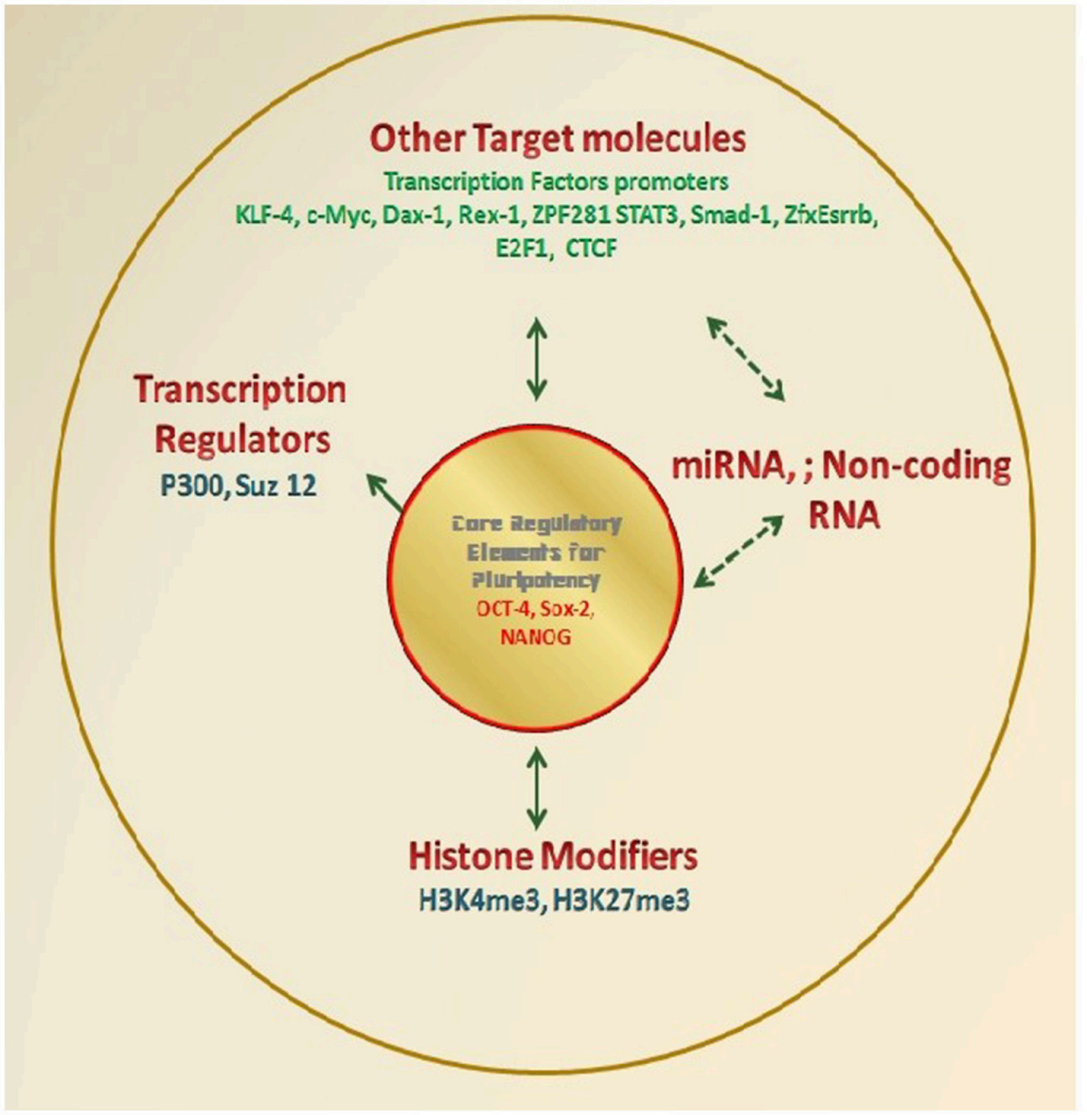
**Molecular diagnostics for determination of Pluripotency**. Various research groups have demonstrated the molecular markers especially TFs which essentially regulate the self-renewal and differentiation potential of PSCs. These factors either directly regulate renewal/ differentiation through their binding to the target gene and therefore regulating target gene expression; or they themselves get regulated and are the target for a number of other transcription regulating elements such as miTNA, Noncoding RNA, Histone modifiers. Various researchers have wisely used these factors as the marker to define the potential of any test cells as an alternate approach to the standard functional assays.

The pluripotency of cells is mainly governed by a number of molecular mechanisms which regulate the expression of genes responsible for the maintenance of primitive stages and repression of differentiation. There are a number of key molecules which sustain the self-renewal, but inhibit the genes for differentiation or keep them silent till external stimuli inducing differentiation activates them. Few important TFs are playing the crucial role in the maintenance of pluripotency, and it is their expression level which defines the pluripotency in various types of ESCs/EpiSCs (Figure 2).

For example, the basal four reprogramming factors OCT4, SOX2, KLF4, and c-MYC (OSKM) were found to be able to induce somatic cells reprogramming and are often correlated with the pluripotency state of any cell population. All of these factors have their own important role in the generation and regulation of pluripotency in the PSCs. For example, OCT4 is the most important reprogramming factor of these four. Loss of OCT4 in embryos lacking Smad2 results into premature differentiation of epiblast which supports the role of OCT4 as an exclusive pluripotency marker and further suggests its crucial role in the development of pluripotency in ICM and its maintenance in ESCs (Waldrip et al., 1998). Similarly, SOX2 is important for various development processes such as maintenance of “*stemness”* in neural progenitor cells and its removal lead to their differentiation (Graham et al., 2003). The main role of SOX2 is the regulation of pluripotency and the determination of cell fate. KLF4 is a mediator of LIF-Stat3 signals and it helps OCT4 and SOX2 in the regulation of NANOG by binding to the promoter of NANOG which also plays a crucial role in the maintenance of pluripotency (Zhang et al., 2010). c-MYC has been responsible for the promotion of active chromatin environment, and enhancement of cell proliferation. It also has an important role in the enhancement of the transition that occurs from the initiation to elongation of the transcription. It has been found to be having an important role in the early stages of the induction as evidenced from the enhanced generation of partially reprogrammed cells, which have not yet turned on the genes for OCT4, SOX2, and KLF4 (Schmidt and Plath, 2012).

Further, it has been reported that some molecular markers such as stage specific embryonic antigen-1 (SSEA1) and alkaline phosphatase get induced in very early stages of reprogramming. Few of the SSEA1+ cell give rise to reprogrammed cells through the activation of OCT4, SOX2, and NANOG (Ho et al., 2011). Many reports demonstrate their particular expression profiles and genetic screening assays to define their essential roles in maintaining pluripotency in both mice and humans (Schöler et al., 1990; Nichols et al., 1998; Avilion et al., 2003; Chambers et al., 2003, 2007; Mitsui et al., 2003; Boyer et al., 2005; Loh et al., 2006; Takahashi and Yamanaka, 2006; Masui et al., 2007; Wang et al., 2012).

OCT4 interacts with other TFs to activate and repress gene expression in mouse ESCs (Pesce and Schöler, 2001). For example, OCT4 can heterodimerize with the SOX2 (HMG-box transcription factor) to regulate gene expression level of many genes in mouse ESCs (Yuan et al., 1995; Botquin et al., 1998; Nishimoto et al., 1999).

However, the role of NANOG in the induction and maintenance of pluripotency is not very clear. It may not be essential in mouse PSCs (Chambers et al., 2007). The low-level expression of NANOG (or even absent) in mouse EpiSCs indicates that it may not be the essential part of the core regulatory circuitry (Figure 2). However, it is reported to stabilize PSCs and required for the development of pluripotency in ICM *in vivo* (Silva et al., 2009). Its co-localization with OCT4/SOX2 indicates its potential role in the maintenance of pluripotency in both mice and humans. Reports show that the major role is owned by OCT4 in the maintenance and induction of pluripotency while under exceptional circumstances SOX2 and NANOG may either be replaced by other substitutes or removed without losing the pluripotency in PSCs.

Genome-scale location analysis for the identification of OCT4, SOX2, and NANOG (OSN) target genes revealed that they co-occupy a substantial portion of their target genes. Most common target genes encode for the transcriptional factors, and many of them are developmentally important homeodomain proteins (Boyer et al., 2005; Loh et al., 2006). These studies suggested a regulatory model for the maintenance of self-renewal potential and repressing differentiation by cooperative binding of OSN to their promoters and thus auto-regulates their function (Boyer et al., 2005; Loh et al., 2006).

Studies in mouse ESCs to understand regulatory network maintaining pluripotency defined target promoter for a number of TFs including OCT4, SOX2, NANOG (and others, e.g., KLF4, c-MYC, Dax1, Rex1, Zpf281, and Nac1). These results indicated two categories of promoters for the target genes on the basis of the number of TFs bound to them (TFs no <4 indicates inactive or repressed promoter; TFs no. >4 indicates promoter active in the pluripotent state but gets repressed during differentiation). Similar studies have been carried out by using chromatin immunoprecipitation coupled with ultra-high-throughput DNA sequencing (ChIP-seq) for identifying and mapping the locations of some important TFs (including STAT3, NANOG, OCT4, Smad1, SOX2, KLF4, Esrrb, Tcfcp2l1, Zfx, c-MYC, n-MYC, E2f1, and CTCF). Also, identification of 2 transcription regulators p300 and Suz12 also revealed activation of a substantial fraction of protein-coding, miRNA and non-coding RNA genes in ESCs. At the same time, OSN interacts with the promoters of the genes encoding lineage-specific regulators (Chen et al., 2008; Kim et al., 2008).

Parallel studies established histone modification signatures as crucial regulators of the gene expression in mammals. This is further supported by specific roles defined for the histone three lysine 4 and histone three lysine 27 trimethylations (H3K4me3 and H3K27me3, respectively) role in controlling gene regulation in ESCs (Lee et al., 2004; Boyer et al., 2005; Bernstein et al., 2006; Pan et al., 2007). The OSN are defined to bind promoters of many lineages regulatory genes, and thus, both active (H3K4me3) and repressive (H3K27me3) histone methyltransferase activities (a bivalent state) possessed by ESCs is believed to facilitate activation of developmental genes upon exit from pluripotency (Bernstein et al., 2006). This capability of OSN to activate self-renewal maintenance genes, while repressing lineage-specifying regulators (i.e., Differentiation) is a major indicator for the double hallmark features of ESCs.

PSCs are further classified as naïve or primed PSCs, and different molecular markers can be defined to identify these states among various types of PSCs. A set of diagnostic molecular signatures for mouse PSCs that delineate their proximity to the preimplantation ICM or post-implantation epiblast respectively has been proposed amongst the various pluripotent states. These distinct identification markers include X chromosome status in female cells, global levels of DNA methylation, OCT4 enhancer utilization, and expression levels of specific regulators' group defined as “naïve” TFs (for example a select group of TFs; KLF4, KLF2, Esrrb, Tfcp2l1, Tbx3, and Gbx2; Tesar et al., 2007; Bao et al., 2009; Guo et al., 2009; Silva et al., 2009; Dunn et al., 2014).

The set of above mentioned naïve TFs and Nanog are not expressed (or very low expression level) in primed PSCs. They are also capable of resetting primed PSCs in conjunction with naïve pluripotency culture conditions. This reflects a regulatory intersection between the naïve transcriptional network and epigenetic resetting of both the DNA methylome and X chromosome. Mouse ESCs derived from pre-implantation blastocysts exhibit a ground state which is defined as the configuration where the cells grow without depending on any exogenous signaling stimuli, at the molecular level when cultured along with the LIF and small molecule inhibitors of MEK and Gsk3 kinases (2i/LIF conditions). These conditions are proposed to stabilize the diagnostic signatures of pluripotency in ESCs (Ying et al., 2008; Marks et al., 2012; Leitch et al., 2013).

There are distinct molecular diagnostic markers exhibited by ESCs demonstrating their ground state, including the presence of two active X chromosomes in female cells, low levels of DNA methylation, high activity of OCT4 distal enhancer, and naïve transcription factor expression. On the other hand, use of FGF and ACTIVIN supports primed ESC state. While comparing with the naïve ESCs, primed EpiSC is defined to have inactivated forms of X-chromosome in female cells, a higher degree of DNA methylation, high activity of proximal enhancer elements for OCT4 gene and repression of naïve TFs.

There have been studies showing genome-wide DNA methylation maps for different stages of PSCs, and it is proposed that different molecular changes including DNA methylation be highly dynamic during mammalian embryogenesis. These developmental changes of ground state ESCs to EpiSCs (primed) *in vitro* also resembles the changes during *in vivo* maturation of preimplantation epiblast to post-implantation epiblast (Smith et al., 2012; Boroviak et al., 2014).

Furthermore, the fine line existing between naïve and primed states defining markers reveal a heterogeneous distribution of various diagnostic markers among these states. It is evident from the observation that both naïve PS markers (e.g., Chimera formation capability) and post-implantation Epiblast marker (e.g., High DNA methylation pattern) are exhibited by mouse PSCs (Leitch et al., 2013). Similarly, this molecular heterogeneity is observed in EpiSCs which are demonstrated to engraft into posterior epiblast and possess diagnostic markers of post-implantation stage indicating their primed PSC state (Wu et al., 2015). While, region-selective EpiSCs are also reported to harbor high cloning efficiency, indicating their naïve PS like properties. These variable shows the need to redefine the various states and their corresponding diagnostic markers more precisely and that seems to incorporate a greater spectrum of functional and molecular state markers. Similarly, the concept of ground state PSCs appears to be incompletely defined. It is indicated by the existing heterogeneity as demonstrated in gene expression profiles, flow cytometry and replating experiments in single cell studies (Kumar et al., 2014). This heterogeneity demonstrates the coexistence of distinct molecular/functional states in mouse ESCs (Kumar et al., 2014). This inherent metastability of PSCs is further reflected by existing heterogeneity among individual cells. Further, even much homogenous ground state cultures have been reported to bear variable TFs expression profile showing dynamic states of PSCs that is both time and space related (Morgani et al., 2013).

However, detailed information on the origin and consequence of this heterogeneity is yet to be developed and by that time PSC classification shall remain ambiguous due to the dynamic nature of pluripotency defining markers. It is important to be noticed that molecular markers may not strictly define the expected functional development of PSCs. For example, mouse PSCs were demonstrated not to get differentiated (an indication of functional pluripotency) but exhibit all the key molecular features of pluripotency such as expression of core TFs in the absence of DNA methylation and H3K27 methylation activities (Li et al., 1992; Okano et al., 1999; Tsumura et al., 2006; Chamberlain et al., 2008). This indicates that the pluripotency of any cell may only be decided by carefully comparing molecular markers and their capability to perform functionally.

Similar results are not available for the human ESCs which do not grow in the absence of DNA methylation gene DNMT1 and thus shown functional differences from the mouse ESCs (Liao et al., 2015). Further, naïve PSCs are not affected by the epigenetic regulatory factors which play important role in the primed PSC state.

## Assessment of pluripotency by various functional assays

**Figure 3:**
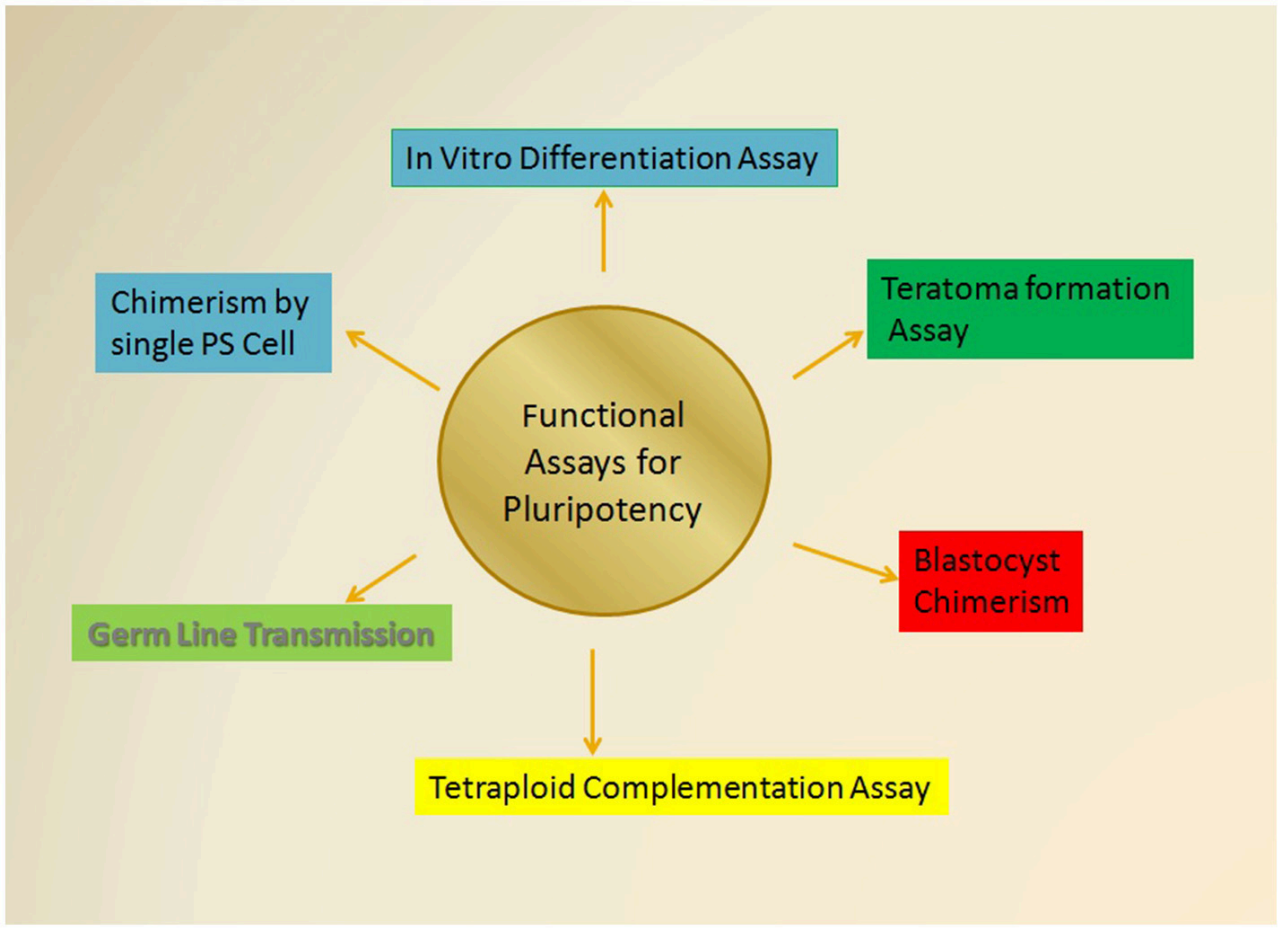
**The Various functional assessment approaches for Pluripotency**. The most stringent tests for the determination of pluripotent cells that includes the various functional assay that essentially rely on the capabilities of test pluripotent cell to self-renew and differentiate into all the three germ cell lineages including ectoderm, mesoderm, and endoderm as mentioned in the text. Some of these assays would also explore the ability of test cells to give rise the whole organism and give rise to a chimeric animal of desired characteristics.

Since the discovery of stem cells various advancements in the study of pluripotency has been evolved from initial embryological experiments to the *in vitro* generation of an array of pluripotent states. Initial studies were done by using murine ESCs (Evans and Kaufman, 1981) that remain an amenable and convenient platform to study various developmental pathways and identification of the major regulating factors of sustenance of pluripotency. Subsequently, development of methods and identification of different other types of pluripotent cells, such as EG cells, EpiSCs, and IPSCs led to the comprehension of the existing methodologies (Figure 3). In 1998, human ESCs were defined for the first time (Thomson et al., 1998) and a wealth of existing information about mouse pluripotent states served as a reference point in defining human pluripotency. Recently, the advent of iPSCs and more advanced technical resources have provided some diagnostic tools for defining pluripotency as discussed above.

The study of various types of PSCs and their capability to give different developmental states has led to the accumulation of many functional assays. These functional assays provide stringent quality assessment tools for the evaluation of pluripotency in both human and mice PSCs (Figure 3). These experimental assays are majorly categorized as follows: (i) *in vitro* differentiation into all three germ layers; (ii) capability to form teratomas; (iii) chimerism of blastocysts; (iv) tetraploid complementation; (v) transmission of germ line; and (vi) chimera formation by using single pluripotent cell.

### *In vitro* differentiation assays

Pluripotency is defined as the ability to give rise to cells from all three germ layers of the body, which may be evaluated *in vivo* (teratoma formation assay), and/or *in vitro*, by inducing the differentiation of the ESCs into all three germ layers. Initially, it was defined via the demonstration of aggregate formation (embryoid bodies) in a hanging drop suspension culture method (Wobus et al., 1984). Since then, various protocols have been published showing the use of small molecules and growth factors to induce differentiation into all three germ layers (Table 1). However, *in vitro* differentiation is considered as the least stringent test to establish the pluripotency.

Generally, *in vitro* differentiation assays are performed by including stem cell culture (e.g., Embryoid bodies) in the media containing a cocktail of differentiation inducing cytokines, morphogens, or chemicals, and after that marker of a specific target, tissues are identified to establish their differentiation (Keller, 1995; Smith, 2001). For example, differentiation of neural tissues (neurospheres) from neural stem cells (Lee et al., 2000; Studer, 2001; Ferrari et al., 2010), cardiac bodies generation from cardiac stem cells and their further differentiation into cardiomyocytes, endothelial cells and smooth muscle cells (Taubenschmid and Weitzer, 2012; Hoebaus et al., 2013) and definitive endodermal differentiation from mouse ESCs (Borowiak et al., 2009; Zhu et al., 2009). Mouse ESCs aggregates are reported to resemble early stages of mouse embryonic differentiation (for 7–8 days) which spontaneously develop into cells of ecto-, meso-, and endodermal origins (Bader et al., 2001). These embryonic bodies are highly useful in studying cellular functions (by electrophysiology) and cell-cell interactions (by using immunofluorescence assays). Whereas, pluripotency assessment in human cells is inherently an intractable problem. As it is described above, pluripotency evaluation in animal (e.g., mouse, rat, primates, etc.) involves their verification through direct means i.e., PSCs are introduced into a developing embryo and their differentiation potential can be directly determined by evaluating the degree of chimerism and/or by determining the organism's viability that is derived from *in vitro* stem cells (Nagy et al., 1993). This may not be applicable in humans because of ethical reasons which would not allow these types of stringent experimentation in the human system; thus indirect evidence through biomarkers and correlative measures of differentiation potential are the next best level of evidence for human stem cell pluripotency. There are many pluripotency determining markers being used for human cells which are chosen by the existing evidence from other species. Defining pluripotency in human system may be erroneous due to: (1) availability of small number of markers and their use would erroneously validate potentially abnormal stem cells which may also express the similar limited set of markers as in normal stem cells, (2) despite some striking orthologous similarities, the parallel comparison of mechanistic insight from animals to the humans for the determination of pluripotent features is problematic due to species-specific differences in the cultured pluripotent phenotypes. In addition, there are evolutionary structural variations in the transcriptional communicating networks of human and animal cells. There are few techniques such as, *in silico* genome-wide methods (e.g., whole genome transcriptome profiles in combination with complex biomarker models) which may be useful in defining the ideal phenotype on a global scale (Skreb et al., 1971; Müller et al., 2008, 2011; discussed in a later section).

### Teratoma formation assays

This test is commonly used for defining the pluripotency in mammals and being used for many decades (Stevens and Little, 1954; Stevens, 1958, 1970; Solter et al., 1970; Skreb et al., 1971; Evans and Kaufman, 1981). Moreover, it is a widely accepted method in stem cell research and banking (ISCBI, 2009; Gertow et al., 2011; Wesselschmidt, 2011). The process can be summarized as the injection/infusion of test cells (expected to be pluripotent) in animal models (mostly mice) into various anatomical sites such as subcutaneous, intramuscular, under the capsule of the kidney, or intra-testicular, of immunocompromised mice and assessment of their potential to develop a tumor. The pluripotency of the test cells is judged on the basis of their ability to develop a tumor with cells exhibiting characteristics of all three germ layers, namely ectoderm (nerve/skin), mesoderm (bone, cartilage/muscle), and endoderm (liver/gut; Brivanlou et al., 2003).

Although, used regularly and frequently, there have been various issues related to the significance of this assay to define pluripotency. The teratoma assay needs to be standardized regarding graft site, the age of animal/mice, a number of cells or graft size and the preparation cell details for various pluripotent cell lines. The formation of teratomas is invariably influenced by all these factors (Brivanlou et al., 2003; Hentze et al., 2009; Wesselschmidt, 2011). Recently, a systematic evaluation of some of these factors for two ESC lines has been proposed (Gropp et al., 2012). Further, the assay is also regarded as time, cost and labor intensive and raises ethical concerns (induce pain and suffering of the animals used) for the utilization of the assay.

Apart from these issues, there are other technical drawbacks such as inefficient histological analysis of teratomas, which may lead to counting even incompletely reprogrammed cells that generate masses with a superficial resemblance to teratomas yet lack terminal three-germ-layer differentiation (Chan et al., 2009). Also, there is a need for lineage tracing or analysis of donor cell-specific marker to distinguish between host and donor cells. This is important since most of the cell preparation uses matrices or scaffold materials which can elicit inflammation or foreign body reaction leading to the misinterpretation of tissue differentiation (RIKEN, 2014).

### Chimeras of blastocysts

The term chimera (originated from Greek Xíμ*αιρα* “she-goat and sometimes named monster,” a fire-breathing creature bearing body parts of three different animals, including a lion, a serpent, and a goat) referred to a single organism generated from the fusion of two (or more) genetically distinct cells from a different origin. For most of the researcher generation of mouse embryonic chimeras works as a well-established tool for determining cell lineage commitment and pluripotency. Various successful reports demonstrating experimental procedures for chimeras' generation by combining two or more preimplantation embryos or, by introducing test cells (ESCs) into a host embryo. This Chimera production technique gained popularity for designing and generating knockout or knock in mice by using genetically modified ESCs.

Initially (the 1960s), experimental mouse chimera were generated by aggregating two or more whole 8-cell embryos that give rise to normal-sized mice consisting tissues with cells from both the parental embryos (Tarkowski, 1961; Mintz, 1962). Mouse chimera production was also reported by injecting isolated ICM cells into a host blastocyst cavity (Tarkowski, 1961; Gardner and Munro, 1974; Bradley et al., 1984). Later on, ESCs PGCs (Matsui et al., 1992) generated by somatic cell nuclear transfer ESCs (ntESCs) (Wakayama et al., 2001) and iPSCs (Okita et al., 2007) were also demonstrated to produce mice chimeras. Further, mouse chimeras are also reported by using tetraploid host embryos.

Apart from these conventional methods (e.g., aggregation of embryos or embryonic cells derived from the same developmental stage), mixing of embryonic cells from different developmental stages can also result in the chimeric offspring (Gearhart and Oster-Granite, 1981; Nagashima et al., 2004). For example, isolated ICMs are shown to produce chimeras when injected into the blastocoelic cavity of host blastocysts, and at the same time when introduced in 8-cell or morula stage embryos (Butler et al., 1987; Polzin et al., 1987; Roth et al., 1989; Nagy et al., 1990; Picard et al., 1990).

In order to identify the contribution of the parental cells in chimeric organism various genetic, biochemical and phenotypic markers are developed. For example, the distinct coat color pattern (a different strain of mice), electrophoretic variants of the housekeeping enzyme glucose-6-phosphate isomerase has frequently been used in earlier studies. In addition, DNA satellite markers (strain-specific and first generation genetic marker; Rossant et al., 1983) are well-reported for cell lineage tracking based on *in situ* hybridization of histological samples that would strike out the different origin of the chimera cells.

Histochemical staining methods to detect β-galactosidase enzyme (*E. coli* lacZ gene and the epifluorescent microscopic methods for the detection of green fluorescent protein are widely used markers. There are new genetic markers also such as microsatellites (short tandem repeats) being employed by a few researchers (Tachibana et al., 2012). Experimental embryonic chimeras have been reported in a number of species, including sheep (Tucker et al., 1974), rats (Mayer and Fritz, 1974), rabbits (Gardner and Munro, 1974), and cattle (Brem et al., 1984) and more recently in nonhuman primates (Tachibana et al., 2012). Despite the demonstration of ESCs or ESC-like cells expressing pluripotency markers from several species, the germline chimeras are reported only for the mouse and rat cells. Firstly, rat ESCs expressing typical pluripotency markers (e.g., differentiate into derivatives of all three germ layers) were reported to contribute germline chimeras (Li et al., 2008) and their reintroduction into early cleaving embryos was capable of inducing the chimera formation (Li et al., 2008). Recent advances in iPSC technology have been useful to generate PSCs from many species, including rats (Liao et al., 2009), cattle (Han et al., 2011), sheep (Liu et al., 2008, 2012), rhesus monkey (Liu et al., 2008), and pig (Ezashi et al., 2009; Wu et al., 2009). Recently, porcine iPSCs has been shown to exhibit chimera generating potential with multiple tissue contribution for the three germ layers (West et al., 2010). Generation of naïve-like porcine iPSCs has been reported with the capabilities of contributing many organs in the fetal chimera (such as head, atrium, ventricle, liver, brachial arch, limb bud; Fujishiro et al., 2013). In humans, embryonic chimerism is shown to occur by spontaneous aggregation of two different zygotes/embryos and due to lack of detectable features of chimerism, most of these conditions may not be diagnosed. Therefore, chimerism in human is generally reported by studies from either developmental anomalies or genotype/sex discordance (Boklage, 2006; Mascetti and Pedersen, 2016).

Experimental human chimerism has been successfully demonstrated by aggregation of fertilized embryos with unfertilized embryos (parthenogenesis or androgenetic) or by aggregation with the fertilized second polar body (Strain et al., 1995; Malan et al., 2006). Thus, these studies have firmly established the method for chimera production as a powerful tool, and it is conceivable from a number of experimental reports that high-quality PSCs would support the generation of high-grade chimera. This is assessed by extensive colonization of all embryonic cell lineages/tissues including germ line. On the other hand, comparatively reduced embryonic viability and small frequency of chimerism would indicate the relatively little potency of the test cells.

### Tetraploid complementation assays

The tetraploid complementation assay is a technique used to construct genetically modified organisms, the study of mutation related consequence of embryonic development, and in the study of PSCs (Nagy et al., 1993; Kang et al., 2009).

This assay is based on the fact that the different lineage potency is displayed by the two cellular constituents of the (ESC ↔ Tetraploid embryo) chimera. On one hand, the ESCs are developed into all structures in the fetus, while the tetraploid cells produce the extraembryonic primitive endoderm tissue and the trophectoderm.

One can carefully select genotypically different ESCs and the tetraploid embryos (mutant versus wild type) to achieve completely segregated cells (mutant cells vs. wild-type cells) into two distinctive tissue compartments of the conceptus. The method involves the production of a tetraploid cell (4n chromosome) by fusing both the cells of a two-cell stage embryo by applying an electrical current. This results in a tetraploid cell which can divide and give rise to similar 4n daughter cells. Although, this tetraploid embryo may develop up to the blastocyst stage and can implant in the uterus wall generating extraembryonic tissue (placenta) but it usually cannot be developed into proper fetus due to its abnormal genetic composition. The tetraploid complementation assay would use a tetraploid embryo (either at the morula or blastocyst stage) that is mixed with normal 2n ESCs (i.e., Test cells) from a different organism to develop into the fetus. Fetus cells are exclusively derived from the ESC (test cells) while the tetraploid cells contribute to the generation of extra-embryonic tissues. Therefore, this ESC-tetraploid embryo chimera becomes a useful experimental tool to test the essentially required genes to function in the embryonic versus extra-embryonic tissues for the development of the concepts. This is one of the most stringent assays to check the pluripotency and developmental ability of the ES or iPSCs. The study of the manner in which development of an embryo occurs helps to determine the effects of a specific allele (wild type/mutant) on the development of the organism. Abnormal development of the embryo from mutant tetraploid cells/wild type ESC composition would indicate defects in the extra-embryonic tissues that are likely to be imposed by the mutation of the extra-embryonic endoderm and the trophectoderm only. Whereas, the development of an abnormal embryo from mutant ESCs (in the epiblast-derived tissues) and wild-type tetraploid cells (in the extra-embryonic tissues), would be visible in the embryonic tissues or the extra-embryonic mesoderm.

So, if there is a lethal mutation in the tetraploid cells, the abnormality in the development of the organism would be due to the defects in the extra-embryonic tissues. This allows us to target the specific gene of interest so that the mutation is removed (wild type) and the potential of a liveborn organism can be achieved.

### Transmission of germ line

Germ line transmission is a process in which the ES derived cells of a chimera result in the formation of reproductive cells of the organism. As the germ cells are passed on to the inherited offspring, it is known as germ line transmission. The assessment of iPSCs for their capability for the formation of chimeras contributing to all of the three germ layers, trophectoderm and the gonads is done by studying the transmission of the germ line. iPSCs possess *in vitro* differentiation potential and morphological immunoreactivity but their plasticity still needs to be tested for the formation of chimera which is an important assay for pluripotency as the iPSCs derived from the transduction of Oct4, Sox2, Klf4, and c-Myc possessed many pluripotent characteristics but were unable to form chimeras which indicates partial reprogramming (Esteban et al., 2009). The capability of the chimera to breed and give rise to viable all-donor pluripotent stem cell-derived offspring is a direct indicator of their success and complete chimerism. The generation of live chimeric offspring validates the potential of the iPSCs for the application in stem cell therapies.

Technically, donor derived test PSCs are integrated into all tissues of the later stages of viable embryos and those results in the adult mice with chimeric tissue throughout their body including germ line tissues. Further breeding of these chimeric animals would lead to all donor PSCs-derived offspring (germ line transmission) which is a robust indicator of chromosomal integration and functional pluripotency.

### Chimera formation by using single pluripotent cell

Chimeric or entirely embryonic stem (ES) cell-derived mice may be produced by using Test cells into diploid (2n)/tetraploid (4n) host blastocysts respectively to demonstrate their pluripotency. As described by a number of successful experiments with ~10–15 ESCs can be injected into the host blastocyst. Since pluripotency stringently refers to the property of a single cell to produce chimeras due to its widespread contribution. Analysis of single PSCs-derived embryo would be of great significance in determining clonal capacities of the test PSCs (Wang and Jaenisch, 2004). However, single-cell chimerism and tetraploid complementation assays are referred almost definitive tools for determining pluripotency of any cell population, but as they suffer from higher failure rates a researcher may not be able to choose them as obvious tools.

## Defining human pluripotent cells on the basis of molecular and functional diagnostic tools

Human pluripotent cells (conventional) may be defined on the basis of various molecular markers and functional assays developed as “Primed” embryonic stem cell state. Since it is shown to utilize the OCT4 proximal enhancer more preferentially, and significantly high levels of DNA methylation. It is reported to have inclined tendency to have an inactivated X chromosome in female cell lines (Hanna J. et al., 2010). There are reports regarding the assessment of human “naïve” PSCs by blastocyst chimerism (Takashima et al., 2014; Theunissen et al., 2014; Gafni et al., 2015). Despite ethical constraints on using human ESCs in these studies limited experiments done with both primed and altered human PSCs, which showed a little contribution in chimera production on their infusion into mouse pre-implantation embryos (Takashima et al., 2014; Gafni et al., 2015).

However, studies done by using primate naïve iPSCs have resulted to significant cross-species blastocyst chimerism. These primate naïve iPSCs were injected into mouse blastocysts resulting to the clonal contribution to the solid tissues (Fang et al., 2014).

Further studies to demonstrate interspecies chimerism by using primate ICM cells have not been successful in generating blastocyst chimerism showing their different properties from mouse ICM cells which readily produce chimeras (Tachibana et al., 2012). Chen et al. described low-grade contribution to all three germ layers by using altered primate PSCs (Chen et al., 2015). But the more stringent test like high-grade contribution and germline transmission are yet to be demonstrated to establish naïve pluripotency in primate ESCs.

The most of these studies indicate the distinct behavior of primate PSCs in chimeras studies. Therefore, interspecies chimerism of human cells into mouse embryos would require additional validation before its routine uses to assess human stem cell potency. Recently, hiPSCs and hESCs have been demonstrated to contribute to the normal mouse development by using a stage-matching approach which involved transplantation of hiPSCs/hESCs into gastrula-stage embryos. Both hiPSCs and hESCs were reported to form interspecies chimeras with high efficiency (Mascetti and Pedersen, 2016). The hiPSCs/hESCs well colonized the embryo and differentiate into all the three primary tissue layers. This tissue-specific fate post-transplantation defined the potential of hPSCs to possess pluripotent characteristics and provided mush awaited functional evidence that human-mouse interspecies developmental competency may be useful in defining their pluripotency by carefully matching their stage of development.

The human stem-cell potency is also assessed by comparing transcriptional and epigenetic molecular markers with the pluripotent cells in human preimplantation embryos. As discussed in earlier sections, the degree of DNA methylation and demethylation/remethylation in various PSCs is widely accepted as a diagnostic signature for pre-implantation and germline developmental stage derived cell in mammals (Deng et al., 2009). There are reports of studies revealing the degree of genomic methylation (hypomethylated vs. hypermethylated DNA) which shown hypomethylated genome human pre-implantation embryonic cells. Further, reports depicted a remethylated genome in ICM derived cells and maintenance of hypermethylated genome that is similar to mouse primed PSCs in establishing human ESCs (Guo et al., 2014; Smith et al., 2014). The epigenetic resetting is controlled by distinct regulatory molecules and their interactions with each other in various states of PSCs. Studies in mice naïve PSCs have highlighted a prominent role of TFs such as KLF4, VTFCP2L1, ESRRB, TBX3, and GBX2. Studies demonstrating single-cell RNA sequencing (RNA-Seq) analysis of human preimplantation embryos (epiblast) and hESCs to define gene expression signatures shown significant differences in their transcriptomes (1498 differentially expressing genes). Human preimplantation epiblast cells also express these molecules. On the other hand, hESCs did not express them showing similarities with mouse EpiSC states (Yan et al., 2013). A simple comparison of the expression levels of specific TFs in mice originated naïve cell with the human preimplantation epiblast cells may not be sufficient to define their similar state of development since some of these factors (e.g., KLF4) are absent in human epiblasts.

Further, the interspecies comparison of other molecular markers such as X chromosome inactivation timing and epigenetic erosion in prime human ESCs has raised the complexity (Silva et al., 2008; Okamoto et al., 2011; Anguera et al., 2012; O'Leary et al., 2012), and identification of hES (ground state or naïve PSCs) by comparing these molecular criteria with the mouse ground state PSCs is not completely acceptable and more specific marker may yet to be demonstrated for human PSCs. While these discrepancies are increasingly being observed by various research groups, a new concept of a metastable naïve state of pluripotency is being suggested (Chan et al., 2013; Valamehr et al., 2014; Ware et al., 2014). The uncertainty about the role of X chromosome inactivation is further reflected by studies from PSCs generated by Jaenisch and Smith Laboratories showing expression of mouse ground state specific TFs (Silva et al., 2008; Okamoto et al., 2011; Anguera et al., 2012; O'Leary et al., 2012; Takashima et al., 2014; Theunissen et al., 2014; Gafni et al., 2015). Declined DNA methylation pattern exhibited by PSCs derived by Smith laboratories indicated their embryonic preimplantation state, but other molecular markers such as activities of OCT4 distal enhancer, and limited information regarding the transgene-independent cell lines' characterization raise ambiguities about the stability of the reset state in these human pluripotent cells (Takashima et al., 2014). Therefore, due to these uncertainties among various molecular markers to define the exact developmental state and hence their pluripotency would be possible only after the direct derivation of ground state ESCs from human embryos. These should be accompanied by comprehensive experimental information regarding the transition of these cells from totipotent to pluripotent states in human and primates.

## Bioinformatics and computer-based methods for pluripotency determination

As described in earlier sections PSCs may be useful in various ways to understand human developmental stages, and personalized regenerative medicine for most of the incurable diseases. However, the direct functional assessment of their propensity is cumbersome and even may not be feasible every time. There are efforts reported from various research groups regarding the development of high-throughput production of human stem cells for use in regenerative medicine and standardization of pluripotency assays by deploying a number of approaches that promise to improve unbiased prediction of the utility of both human-induced PSCs and ESCs by using bioinformatics and gene expression profiling.

### Genomic analysis of PSCs

This is first method defined to evaluate the pluripotency of PSCs by gene expression profiling through DNA microarrays. It applied global molecular analysis approach for mapping the transcriptome of PSCs (Sato et al., 2003; Sperger et al., 2003; Bhattacharya et al., 2004; Suárez-Farinas et al., 2005) and has become a standard assay of pluripotency in many studies.

There are a number of algorithms that have been used to classify cell lines into similar transcriptional states. Muller et al. demonstrated a computational way (“Stem Cell Matrix”) that allows the user to the classify cultured human stem cells in various contexts of pluripotency, multipotency, and differentiated cell types and thus, PSCs may be distinguished from other stem cell types/differentiated cell types (Müller et al., 2008). There has been significant progress in the way these methods are being applied to define more subtle differences in PSCs. The earlier comparisons of iPSCs and ESCs indicated statistical differences between them (Maherali et al., 2008; Chin et al., 2009; Soldner et al., 2009), and which was visible even in their later stages with a significantly reduced level. Recent advancements established global similarities with small differences between iPSCs and hESCs (Marchetto et al., 2009; Bock et al., 2011; Lister et al., 2011) such as changes in gene expression signature in miRNA and long intergenic noncoding RNA along with mRNA (Lakshmipathy et al., 2010; Loewer et al., 2010; Stadtfeld et al., 2010). However, a more comprehensive study would be required to define the reasons behind these variations which may be due to differences in growth parameters/conditions, laboratory-to-laboratory variation (Newman and Cooper, 2010), heterogeneity in iPSC quality (Maherali et al., 2008), or small sample sizes (Chin et al., 2009).

One must be interested in developing such a method that may be useful in identifying the pluripotency of any individual cell or cell population. Since the gene expression profile is highly variable among different pluripotent cells, it may be difficult to establish such a signature expression profile, especially when the sample size used in these gene expression studies is relatively small and may not be sufficient to find consistent small observable differences among different PSCs (Bock et al., 2011). Advancement in the availabilities of curated databases with sufficiently large no. of samples should ensure the reliability of these approaches.

### Pluritest

Pluritest presents one of the excellent examples that can quantify the extent to which experimental methodologies may perform in order to convert a parental cell toward the target cell (Müller et al., 2008). Since the assessment of the continuously expanding larger data sets by the researchers through the newer techniques such as machine learning may be helpful in classifying the various types of PSC lines available to the researchers. The Pluritest makes the exact same comparisons by using an algorithm that relies upon the use of training sets containing sufficiently large dataset including huge numbers of (undifferentiated, differentiated, normal, and abnormal) human stem cell lines and tissues. These databases with larger sample sizes are used to develop bioinformatics models for identifying stem cells' pluripotency on the basis of gene expression measurements done by DNA microarray (Müller et al., 2011). The bioinformatics model for these assessments is generated by using two important vectors that are calculated to categorize (i) pluripotent cells vs. differentiated cells, and (ii) abnormal vs. normal gene expression signature profiles. This calculation utilizes a large training set of almost 500 samples curated for microarray data quality and then consists results from microarray data originated from the studies done on various types of pluripotent cells e.g., hESCs, germ cell tumors, primary cell lines, and somatic tissues microarray analysis. Pluritest algorithm has been reported to distinguish the different test samples of germ cell tumors from hESCs independently as well as distinguish reprogrammed from partially reprogrammed iPSCs. Further, it is also useful in identifying parthenogenetic stem cell lines and separating them from hESCs which are assumed to be a manifestation of differences at imprinted loci. This ability could be helpful in distinguishing between abnormal and normal samples and also in their categorization as undifferentiated/differentiated cell stage.

The algorithm has also been reported to be useful for iPSCs characterization (MacArthur et al., 2012; Mariani et al., 2012; Nazor et al., 2012). Similar models for mouse ESCs are also developed showing their ability to calculate the reprogramming efficiency in response to NANOG overexpression (MacArthur et al., 2012; Mariani et al., 2012; Nazor et al., 2012). The algorithm may find the further application, and various other types of databases (e.g., epigenetic status or stem cell lines) may be examined to define valuable information (Williams et al., 2011). Although, the efficiency and sensitivity of these algorithms to determine abnormalities e.g., copy number variations or translocations is yet to be established. One approach to improving this quality may be the inclusion of genomic integrity tests and improving the variability of the training dataset used to construct the model.

### Comparison of epigenetic profiles of PSCs

Despite inconsistencies monitoring epigenetics may be sensitive enough to define small changes and a more elaborated definition for their functional consequences may be developed. For example, together methylation mapping and gene expression signatures methodologies by these sophisticated algorithms may help in generating robust tools to infer the cell state. Bock and colleagues shown minor yet significant differences in DNA methylation and gene expression pattern in some IPS cell lines but not in ESCs lines through a number of statistical tests against preexisting datasets (Chin et al., 2009; Doi et al., 2009; Stadtfeld et al., 2010; Bock et al., 2011; Theunissen et al., 2014). As described in their studies by using a support vector machine learning algorithm to classify the data, including both from DNA methylation and gene expression data obtained from ESCs lines (20 lines) and iPSCs lines (12 lines) this method efficiently classifies ESCs cell lines. However, results with the iPSCs were of moderate importance only when trying to classify iPSCs lines. In brief, iPSCs specific gene signature was identified with great accuracy (81%) and specificity (91%) but with moderate sensitivity (61%). Considering the size of training datasets used (for combined methylation and gene expression) in these studies a significantly bigger dataset was used in similar methodologies employed by Pluritest and its larger data set for training set development in this method may also improve the predictions.

### Newer methods to analyze the differentiation potential of PSCs: the scorecard approach

Since the right selection of most suitable undifferentiated cell (PSCs) is a key to determine the success of developing a desired cellular lineage for both the clinical and research purposes. Presently used labor intensive methods e.g., teratoma formation assay etc. are both time consuming and expensive. The different *in silico* approached discussed so far mostly focus on the identification and characterization of the status of differentiation in PSCs and may not be able to predict their ability to differentiate into various required lineage cells. Bock et al. (2011) have tried to fill this gap by demonstrating a method which predicts this ability of PSCs by using combined gene expression data and epigenetic measures with *in vitro* differentiation assays. In their report, firstly a deviation scorecard is generated to evaluate existing data for DNA methylation and gene expression profiles. These profiles are relative to a set of reference standard has lines which identify deviated lines as reported by outlier detection methods.

This analysis would result in a number of genes (outliers) that may be screened for their probable role in respective functional assay's performances (Boulting et al., 2011). For example, their group assessed the data to identify genes associated with an aberrant function for motor neuron when iPSCs is directed to get differentiated into neuronal lineages. These studies could highlight one putative gene GRM (glutamate receptor in a motor neuron), and thus, the particular cell line may be ruled out on the basis of this test result.

Further, a quantitative embryoid body assay through high-throughput counting methods was used to develop quick methods of highlighting the differentiation potential for PSCs and develop a respective scorecard for the same. In a non-directed embryoid differentiation assay (20 ESCs and 12 iPSC) RNA analysis was done to probe the expression level of 500 marker genes by Bock and colleagues. The results obtained from these studies were used to generate a quantitative gene expression profile from reference hESCs line derived embryoid bodies. As described in their reports, the lineage scorecard successfully detected and classified iPSCs lines based on their ability to differentiate into ISL1-positive motor neurons in direct differentiation assays.

In brief, prediction of differentiation fate in iPSCs could be possible by the integration of the multiple high content functional assay data. The linear scorecard approach is also beneficial in predicting the possible ability of a particular cell line into specific lineages on induction with appropriate factors/culture environments through the selection of specific gene sets and recalibration to reference standards. With the enhancement of data (number of lines screened increases) identified potential of this approach for the most frequent gene expression and epigenetic aberrations may be achieved. In addition, frequent use of these methods may benefit by reducing the cost and time taken by conventional assay to decide the most appropriate lines for a particular set of experiments or regime.

## Conclusion

It is clear from the available information that pluripotency is a dynamic state, and a large number of molecular factors essentially regulate the fate of any developing cell. There are many different types of molecular markers such as TFs, their target gene promoters, miRNAs, noncoding RNAs, histone modifiers proteins, and their corresponding gene expression, which are being scrutinized by the global research community to define the level of pluripotency. However, there have been some discrepancies among these methods, and the reliability and authenticity of these diagnostics markers may be liable to vary for different cell types being evaluated by the researchers. Since these molecular diagnostic molecules are yet to be optimized for their ability to become more sensitive and accuracy of the prediction made on the basis of the various bioinformatics approaches would also be improved. Since then, only the functional assays of various types, e.g., teratoma formation assays, blastocyst chimerism, tetroid complementation assays, etc. would remain the gold standard for the determination of pluripotency. Recent advancements in the technology to get pluripotent cells by reprogramming (iPSCs) and more robust molecular techniques such as CHIP-seq, microarrays and enhanced computing methods, together all these would further enhance our understanding of the core regulatory elements essential for the maintenance and induction of pluripotency in various types of cells. Further, the capability to predict the most obvious outcome by *in silico* tools when a PSC line is allowed to differentiate would improve the clinical outcome in near future.

## Author contributions

All authors listed, have made substantial, direct and intellectual contribution to the work, and approved it for publication.

## Funding

INSPIRE Faculty award is a prestigious award given to the candidates working in all the scientific fields. The award supports young and upcoming scientist with a fellowship and research grant for a max period of 5 years. VS is thankful to the DST and INSA for providing him an opportunity to work independently and develop as a scientific personnel.

### Conflict of interest statement

The authors declare that the research was conducted in the absence of any commercial or financial relationships that could be construed as a potential conflict of interest.
